# Data for 3D reconstruction and point cloud classification using machine learning in cultural heritage environment

**DOI:** 10.1016/j.dib.2022.108250

**Published:** 2022-05-10

**Authors:** Massimiliano Pepe, Vincenzo Saverio Alfio, Domenica Costantino, Daniele Scaringi

**Affiliations:** Polytechnic of Bari, via E. Orabona 4, Bari 70125, Italy

**Keywords:** UAV photogrammetry, Point cloud, Cultural heritage, Random forest, Machine learning, Classification

## Abstract

Unmanned Aerial Vehicle (UAV) photogrammetry, thanks to the development of Structure from Motion (SfM) and Multi-View Stereo (MVS) algorithms, allows the generation of dense point clouds, capable of representing three-dimensional objects and structures in a detailed and accurate manner. In addition, the possibility of associating more semantic information through automatic segmentation and classification models, becomes of fundamental importance in the field of development, protection and maintenance of Cultural Heritage (CH). With the developments in Artificial Intelligence (AI), classification algorithms based on Machine Learning (ML) have been developed. In particular, the Random Forest is used in order to perform a semantic classification of the point cloud generated by UAV photogrammetry and Global Navigation Satellite Systems (GNSS) survey of a structure belonging to CH environment. Indeed, this paper describes the images collected through a UAV survey, for 3D reconstruction of Temple of Hera (Italy) based on photogrammetric approach and georeferenced by the use of 8 Ground Control Points (GCPs) acquired by GNSS survey. In addition, the shared dataset contains the point cloud and data for classification using Random Forest algorithm.

## Specifications Table

**Table:** 

Subject	Data Science
Specific subject area	Applied Machine Learning, Geomatics Engineering, Computer Vision
Type of data	Images, Text file, Point Cloud
How the data were acquired	UAV: DJI Mavic 2 PRO Camera: Hasselblad L1D-20c Planning software: Map Pilot Flight monitoring interface: Apple iPad 5 GCPs measurement device: Leica Viva GS12 Data processing software: Leica Geo Office 3D Photogrammetric and modelling software: 3DF Zephyr
Data format	Raw, Analysed and filtered.
Description of data collection	The photogrammetric dataset was acquired using close range photogrammetry technique with high image overlap in order to obtain a 3D reconstruction of the archaeological remains of the temple. The UAV images were processed in a software based on Structure from Motion and Multi-View Stereo algorithms. In this environment, it was possible to build a detailed 3D model. Furthermore, thanks to the use of Global Navigation Satellite Systems survey in static-rapid mode, 8 Ground Control Points were acquired and, consequently, it was possible to georeference and scale the 3D point cloud. In addition, it was added a dataset containing the point cloud divided into 3 portions for Random Forest classification: Training, Evaluation and Test. The first two portions of the point cloud were classified manually, while the third is the one that the algorithm will classify automatically after training. The point clouds, saved in *.txt* format, are organised in columns: the first 3 columns represent the spatial coordinates of the points (X, Y, Z), the next 7 columns represent the geometric features provided as input to the classifier. The eighth column, present only in the case of Training and Evaluation, represents the class of the point. A total of 5 classes were defined: "architrave": 0, "capital": 1, "column": 2, "stylobate": 3, "stereobate": 4. Finally, the file called "Features Index" (*.txt* format) contains in the first line the indices of the feature columns to be used as input to the classifier, while in the second line is present the index of the column representing the class.
Data source location	Tavole Palatine – Temple of Hera City/Town/Region: Metaponto, Bernalda (MT) - Basilicata Country: Italy Latitude and longitude (and GPS coordinates) for collected samples/data: Lat. 40°24′57.76"N; Long. 16°49′0.38"E (WGS84) East: 654138.03 m; North: 4475520.40 m (UTM33N-WGS84 - EPSG:32633)
Data accessibility	Repository name: Mendeley Data Data identification number: 10.17632/ydpn8xnx24.1 Direct URL to data: https://data.mendeley.com/datasets/ydpn8xnx24/1
Related research article	[1] V.S. Alfio, D. Costantino, M. Pepe, 2020. Influence of Image TIFF Format and JPEG Compression Level in the Accuracy of the 3D Model and Quality of the Orthophoto in UAV Photogrammetry. Journal of Imaging, 6(5), 30. https://doi.org/10.3390/jimaging6050030.

## Value of the Data

• The dataset makes it possible to analyse a structure of particular historical and architectural interest and to classify the elements of the 3D point cloud.
• The data are useful for researchers in the field of geomatics, computer vision, archaeology and architecture for: evaluating Structure from Motion and Multi-View Stereo reconstruction algorithms, point cloud processing, semantic 3D reconstruction, virtual tours, virtual and augmented reality applications.
• New techniques and algorithms for the construction of the 3D point cloud and for the semantic classification of the elements of a structure, with special regard to cultural heritage assets.

## 1 Data Description

**Fig. 1:**
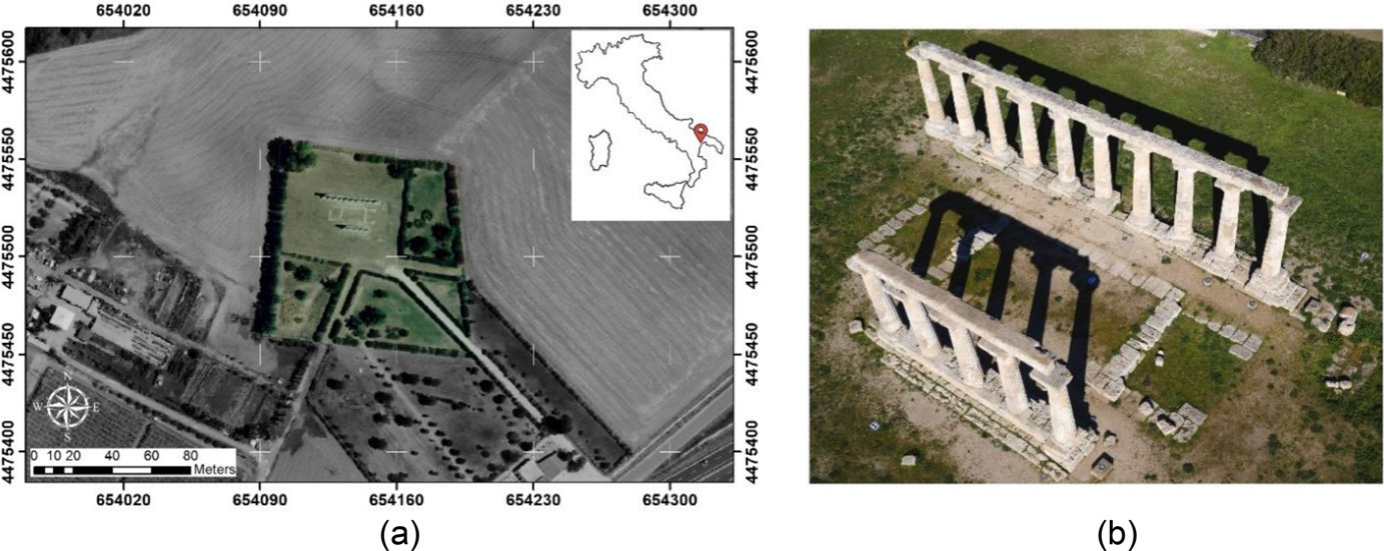
Study area: archaeological site (a), remains of the temple of Hera (b).

This paper describes a photogrammetric survey performed by a UAV platform [2,3] in order to generate a 3D point cloud and its subsequent semantic classification in Cultural Heritage (CH) environment. The test area is located in the archaeological site of Metaponto, Italy and represents the remains of a Doric temple dating from the 6th century before Christ (BC) and dedicated to the Greek goddess Hera, as shown in Fig. 1. (https://data.mendeley.com/datasets/ydpn8xnx24/1).

The photogrammetric dataset consists of 57 images acquired with DJI Mavic 2 Pro UAV (folder dataset “*UAV Images*”, https://data.mendeley.com/datasets/ydpn8xnx24/1*)*. The images were acquired on 01 February 2020 at 10:00 am (local time) in DGN format and subsequently transformed into TIFF format. The photogrammetric dataset was processed in close range photogrammetry [4] software. In particular, Zephyr 3D software was used. In order to georeference and scale the point cloud, it was necessary to determine the coordinates of the Ground Control Points (GCPs) performing a Global Navigation Satellite Systems (GNSS) survey. In particular, the plane coordinates of the GCPs are reported in the repository (folder dataset “*GNSS Survey*”, https://data.mendeley.com/datasets/ydpn8xnx24/1) according to a formatting of the type *East, North, Ellipsoidal Height*.

In this way, it was possible to obtain a colour dense point cloud consisting of 973125 points (folder dataset “*Point Cloud*”, https://data.mendeley.com/datasets/ydpn8xnx24/1*)*.

Subsequently, the point cloud was classified into 5 classes, using Random Forest algorithm. In particular, the dataset “*Point Cloud Classification*” contains text file (“*Box_Evaluation*”, “*Box_Test*”, “*Box_Training*”, “*Features_Index*”), that can be found in the repository (folder dataset “*Point Cloud Classification*”, https://data.mendeley.com/datasets/ydpn8xnx24/1*).*

## 2 Experimental Design, Materials and Methods

### 2.1 Method

The semantic classification of the point cloud can be carried out in two successive steps: the first involves the three-dimensional reconstruction of the site, object or structure taken into consideration starting from images [5] and, subsequently, the application of Machine Learning algorithms for the classification and segmentation of the features [6].

### 2.2 3D reconstruction

For image capture, UAV DJI Mavic 2 PRO developed by DJI Company, Shenzhen, China, was used. The DJI Mavic 2 Pro is a popular consumer UAV (Quadcopter) equipped with a high-resolution colour camera. In fact, the Hasselblad L1D-20c camera features a 1" 20MP sensor that offers better low-light shooting than other drone cameras. Before the aerial survey, GCPs realized in wooden material (black and white chequered or rhombus) and in such a size that they are easily recognised in the images, were positioned uniformly within the surveyed area. The flight planning was designed in Map Pilot for DJI app, according to a grid path, using an overlap and sidelap value respectively of 80% and 60%; in addition, 45° oblique images with an overlap of 60% were added in order to ensure complete coverage of the temple's shaft, capital and architrave.

In order to georeference the model and evaluate the accuracy of the photogrammetric process, GCPs were surveyed with GNSS technique in fast-static mode, using Leica GS12 dual-frequency receiver and using RINEX data obtained from 2 master stations belonging to HxGN SmartNet CORS (Continuously Operating Reference Station). The post-processing of the GNSS data, was performed using Leica Geo Office (LGO) v. 8.2 software. In this way, the coordinates of the GCPs were determined with a plano-altimetric accuracy of less than 0.001 m.

**Fig. 2:**
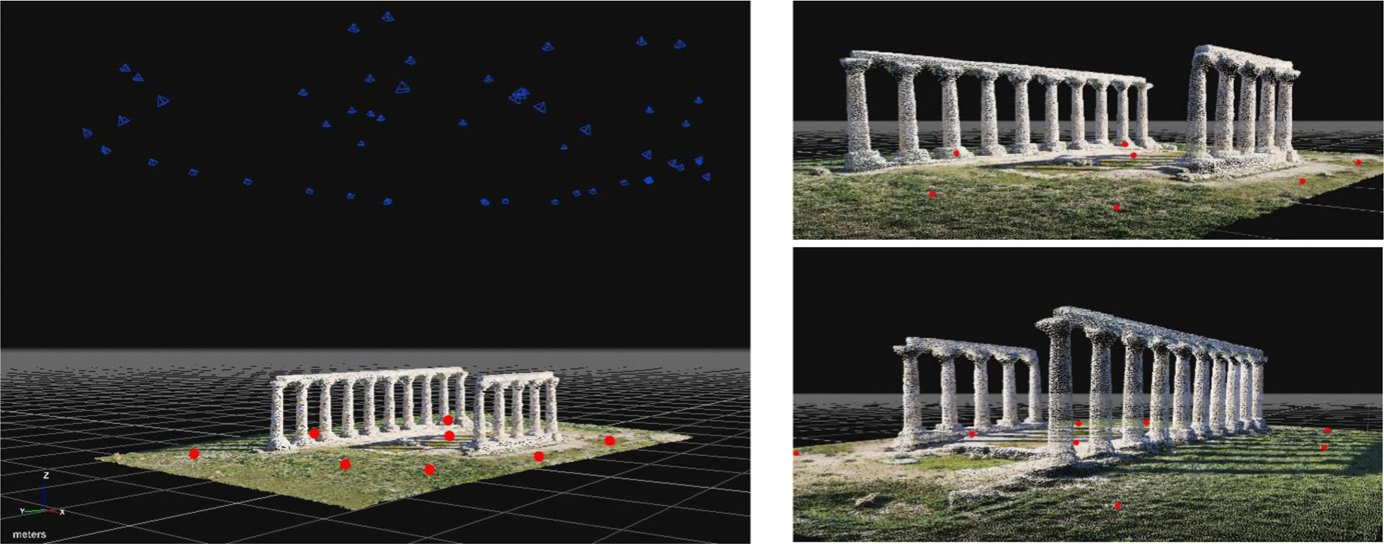
3D point cloud.

The processing of the photogrammetric data was performed in the 3DF Zephyr, which is a photogrammetric software developed and marketed by the Italian software house 3DFLOW. This software is based on Structure from Motion (SfM) and Multi-View Stereo (MVS) algorithms and allows to automatically perform 3D reconstructions. The images were processed by setting "Highest" in the definition of key point density and an “Accurate” mode was chosen in the matching type. Using a special tool implemented in this software, it was possible to import 3D coordinates of GCPs by approximating them to the nearest point of the selected object; if no object is selected, the points are imported directly. The geometric quality of the 3D model was evaluated on several GCPs. The Root Mean Square Error (RMSE) obtained in photogrammetric process was 0.009 m. Furthermore, this process allowed the construction of a dense point cloud of 973,125 points as shown in Fig. 2 (https://data.mendeley.com/datasets/ydpn8xnx24/1).

### 2.3 Point cloud classification

The classification of the point cloud can be performed using different algorithms, such as Hough Transform (HT), Random Sample Consensus (RANSAC) and Random Forest (RF). In particular, the classification of the point cloud of the temple was performed using the RF algorithm [7] and open source software, such as Cloud Compare software and Anaconda (a data science platform developed in Python). RF is a supervised machine learning algorithm that is used in classification and regression problems and consists of constructing decision trees on different samples. To perform point cloud classification according RF algorithm, it was necessary to divide it into three portions useful for the training, evaluation and classification phase. In order to characterise each point, both radiometric and geometric features were combined.

**Fig. 3:**
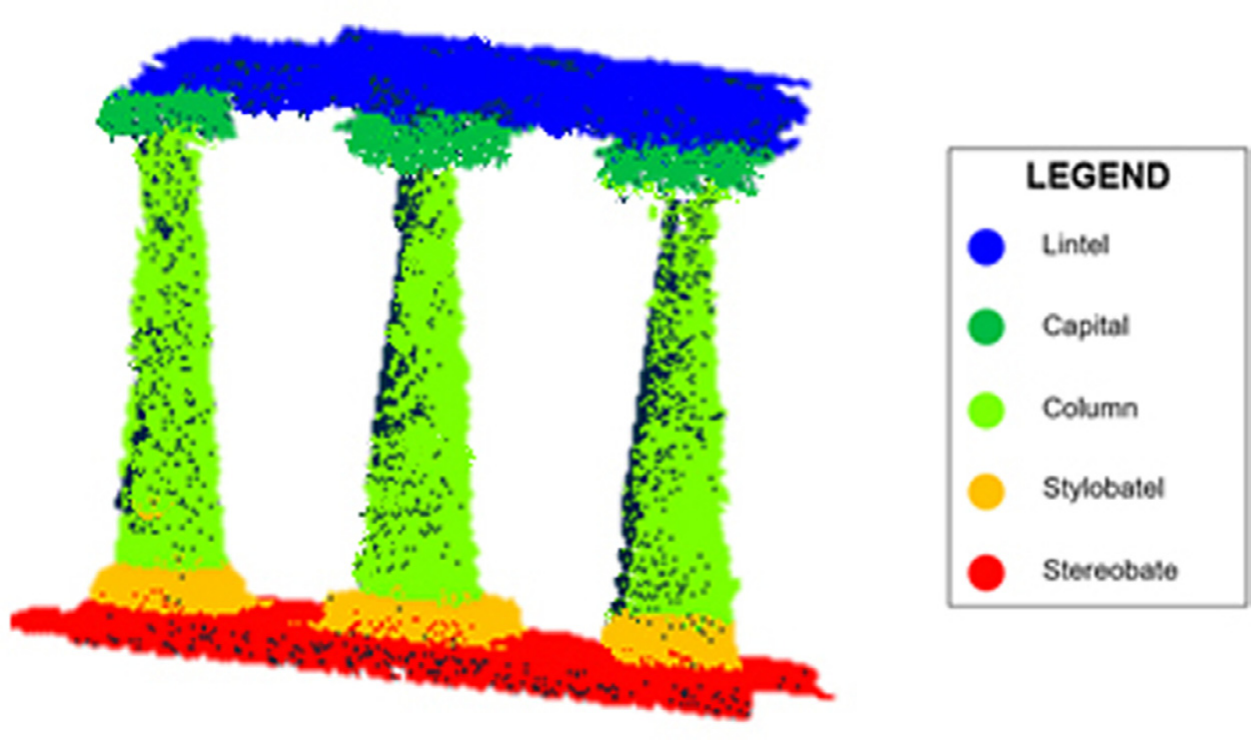
Identification of point cloud classes.

The evaluation of the quality of the point cloud was carried out using four performance indices, such as Precision, Recall, F1 measure and Overall Accuracy [8,9]. Therefore, taking into account the point cloud under investigation, two columns were annotated for the training phase, while the remaining 3 columns were annotated for the evaluations. A total amount of 5 classes were identified, as shown in Fig. 3 (https://data.mendeley.com/datasets/ydpn8xnx24/1).

The other parts of the structure were classified with RF algorithm. Therefore, once the manual annotation phase of the data was finished, it was necessary to evaluate which features to select in order to classify the scene adequately. After this operation, the dataset is used as input in the predictive model.

## Ethics Statements

It is not applicable to this dataset.

## CRediT Author Statement

**Massimiliano Pepe:** Conceptualization, Methodology, Software, Data curation, Writing – original draft preparation, Writing- Reviewing and Editing; **Vincenzo Saverio Alfio:** Conceptualization, Methodology, Software, Data curation, Writing – original draft preparation, Writing – review & editing; **Domenica Costantino:** Conceptualization, Methodology, Software, Writing – original draft preparation, Validation, Supervision; **Daniele Scaringi:** Visualization, Investigation, Conceptualization, Methodology, Software.

## Declaration of Competing Interest

The authors declare that they have no known competing financial interests or personal relationships that could have appeared to influence the work reported in this paper.

## Data Availability

Data for 3D reconstruction and Point Cloud classification using Machine Learning in Cultural Heritage environment (Original data) (Mendeley Data).
